# Transfer of membrane(s) matter(s)—non-genetic inheritance of (metabolic) phenotypes?

**DOI:** 10.3389/fmolb.2024.1347397

**Published:** 2024-03-07

**Authors:** Günter A. Müller, Timo D. Müller

**Affiliations:** ^1^ Institute for Diabetes and Obesity (IDO), Helmholtz Diabetes Center (HDC) at Helmholtz Zentrum München, German Research Center for Environmental Health (GmbH), Oberschleissheim, Germany; ^2^ German Center for Diabetes Research (DZD), Oberschleissheim, Germany; ^3^ Department of Media Studies, Media, Culture and Society, Faculty of Arts and Humanities, University Paderborn, Paderborn, Germany

**Keywords:** extracellular vesicles, glycosylphosphatidylinositol-anchored proteins, inheritance of acquired traits, membrane biogenesis, non-genetic matter, phenotypic plasticity

## Abstract

Glycosylphosphatidylinositol-anchored proteins (GPI-APs) are anchored at the outer phospholipid layer of eukaryotic plasma membranes exclusively by a glycolipid. GPI-APs are not only released into extracellular compartments by lipolytic cleavage. In addition, certain GPI-APs with the glycosylphosphatidylinositol anchor including their fatty acids remaining coupled to the carboxy-terminus of their protein components are also detectable in body fluids, in response to certain stimuli, such as oxidative stress, radicals or high-fat diet. As a consequence, the fatty acid moieties of GPI-APs must be shielded from access of the aqueous environment by incorporation into membranes of extracellular vesicles or into micelle-like complexes together with (lyso)phospholipids and cholesterol. The GPI-APs released from somatic cells and tissues are transferred via those complexes or EVs to somatic as well as pluripotent stem cells with metabolic consequences, such as upregulation of glycogen and lipid synthesis. From these and additional findings, the following hypotheses are developed: i) Transfer of GPI-APs via EVs or micelle-like complexes leads to the induction of new phenotypes in the daughter cells or zygotes, which are presumably not restricted to metabolism. ii) The membrane topographies transferred by the concerted action of GPI-APs and interacting components are replicated by self-organization and self-templation and remain accessible to structural changes by environmental factors. iii) Transfer from mother cells and gametes to their daughter cells and zygotes, respectively, is not restricted to DNA and genes, but also encompasses non-genetic matter, such as GPI-APs and specific membrane constituents. iv) The intergenerational transfer of membrane matter between mammalian organisms is understood as an epigenetic mechanism for phenotypic plasticity, which does not rely on modifications of DNA and histones, but is regarded as molecular mechanism for the inheritance of acquired traits, such as complex metabolic diseases. v) The missing interest in research of non-genetic matter of inheritance, which may be interpreted in the sense of Darwin’s “Gemmules” or Galton’s “Stirps”, should be addressed in future investigations of the philosophy of science and sociology of media.

## Introduction–GPI-APs and membranes, and inheritance?

**FIGURE 1:**
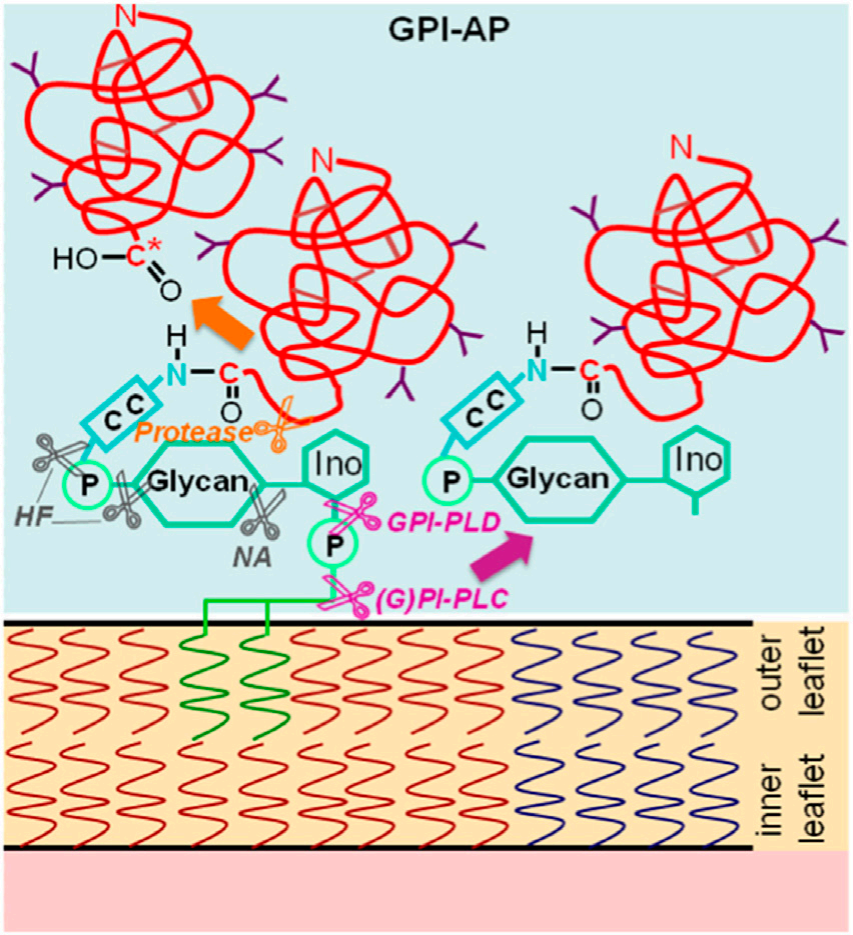
Structure and types of enzymatic (by mammalian, parasitic or bacterial glycosylphosphatidylinositol-specific phospholipases C or D, (G)PI-PLC/D, or proteases that produce a new carboxy-terminus C*) or chemical (by hydrogen fluoride dephosphorylation, HF or nitrous acid deamination, NA) liberation of GPI-APs from PMs into the extracellular space (light blue). C-C, ethanolamine residue bound by an amide bond to the carboxy-terminus of the protein portion and to the glycan core (glycan) via a phosphodiester bridge; Ino, myo-inositol. The model presented is in agreement with the bilayer structure according to Singer and Nicolson (1972) and Nicolson (2013), provided the symbols are being considered as representatives for both lysophospholipids (presumably expressed in areas of upregulated release of GPI-APs) and phospholipids (predominantly expressed in nano- and microdomains of suppressed release of GPI-APs), the latter, however, lacking the connecting glyceryl bridge between two adjacent long-chain acyl moieties, each, for reasons of simplification. The depiction of the GPI-APs, including the major (chemical and enzymic) cleavage sites, follows conventional rules without compromise, with two fatty acyl residues drawn per molecule.

Glycosylphosphatidylinositol-anchored proteins (GPI-APs) are anchored at the outer leaflet of the phospholipid bilayer of eukaryotic plasma membranes (PMs) solely by a glycosylphosphatidylinositol (GPI) glycolipid which is covalently coupled to the carboxy-terminus of the protein moiety via an amide bond (Ferguson and Cross, 1985) (Figure 1).

The glycan core of GPI consists of glucosamine and three mannose residues in typical glycosidic linkage (to which additional carbohydrate side chains may be attached) and is highly conserved from yeast to humans (for a review, see Eisenhaber et al., 2001; Poisson et al., 2007; Fujihara and Ikawa, 2016; Kinoshita, 2020; Müller, 2020; Komath et al., 2023). Currently, about 1.2%–1.5% of all human proteins and 3.9%–5.8% of all human PM proteins, which account for 2,886 entities according to present *in silico* annotation (Albert et al., 2002; Dobson et al., 2015), are thought to represent GPI-APs, as deduced from the presence of both amino- and carboxy-terminal signal sequences (for a review, see Bausch-Fluck et al., 2018; Liu and Fujita, 2020; Jelokhani-Niaraki, 2023). Among the diverse functions attributed to GPI-APs are i) their concentration at micro- or nanodomains of PMs which may subsequently form either (detergent-insoluble) lipid rafts (of low buoyant density) as platform for signaling molecules or invaginations which bud into the cytoplasm as small vesicles or caveolae (for a review, see Müller, 2002; Saha et al., 2016; Müller, 2018), and ii) cleavage of their GPI anchor by (G)PI-specific phospholipases of specificity C or D (GPI-PLC/D) (Davitz et al., 1986; Huang et al., 1990; Stieger et al., 1991; Li et al., 1994; Wong and Low, 1994) (Figure 1), which results in constitutive or stimulus-induced release of the protein moiety into extracellular compartments (for a review, see Fujihara and Ikawa, 2016; Müller, 2020).

However, GPI-APs are not only released from donor cells and tissues as a result of lipolytic cleavage (for instance, see Dolezal et al., 2014; Müller, 2018). In response to certain extracellular stimuli, such as oxidative stress, radicals or high-fat diet, a subset of them is also recovered from extracellular spaces and fluids with the full-length GPI anchor remaining attached at the carboxy-terminus of their protein portions. For this, the fatty acids of the GPI anchor have to be shielded from access of the aqueous milieu (Caseli et al., 2008) by incorporation into i) the outer leaflet of the phospholipid bilayer of extracellular vesicles (EVs), such as microparticles and exosomes (for a review, see Trams et al., 1981; Denzer et al., 2000; Couzin, 2005; Johnstone, 2005; György et al., 2011; Müller, 2012; Hargett and Bauer, 2013; Harrison et al., 2014; Cocucci and Meldolesi, 2015; Clemmens and Lambert, 2018; Jeppesen et al., 2019; Mathieu et al., 2019; Raposo and Stahl, 2019; Couch et al., 2021) or ii) the phospholipid monolayer of lipoprotein-like particles, such as surfactant-like particles or milk fat globules (for a review, see Panakova et al., 2005; Neumann et al., 2007; Eaton, 2008; Müller, 2018) or iii) the interior of so-called micelle-like complexes together with lysophospholipids, phospholipids and cholesterol (for a review, see Müller and Müller, 2023a; Müller and Müller, 2023b) or iv) the binding sites of carrier proteins without the involvement of membranous structures (for a review, see Müller, 2018). In this “Hypothesis and Theory” paper, the possibility is considered that i) the transfer of GPI-APs and other membrane proteins assembled together into certain membranous structures, such as EVs or micelle-like complexes, play epigenetic roles in phenotypic plasticity and the inheritance of acquired traits, and ii) the non-genetic matter involved may be considered as “Gemmules” and “Stirps” in the sense of the “maturational” (or developmental) rather than the “directional” (or informative) theories of inheritance of the 19th century.

At first, some key players, critical (f)actors, and basic mechanisms of the phenomenon of inheritance are presented in a glossary:

**Extra-/intracellular (cytoplasmic) inheritance:** Transfer of non-genetic matter from donor cells or organisms to acceptor cells or organisms via extracellular mechanisms and structures, such as EVs or micelle-like complexes, or via the intracellular distribution of PMs and cytoplasm (i.e., organelles, cytosol).

**EVs**: Small vesicles (30–1,000 nm) that are released into extracellular compartments by almost all eukaryotic donor cells by protrusion of the PMs (microparticles) or exocytosis along the endosomal and secretory pathways (exosomes), either constitutively or in response to environmental factors E), and trigger specific functional changes in appropriate acceptor cells either after fusion with the acceptor membranes (microparticles) or endocytic uptake and endosomal fusion (exosomes). The phospholipid bilayer (membrane) with the integrated transmembrane proteins and GPI-APs of the EVs enclose soluble proteins and (small, circular, messenger) RNA, which also originate from the donor cells. The use of EVs for diagnostics is currently being investigated for a variety of diseases (“liquid biopsies").

**Intercellular inheritance:** Transfer of genetic and non-genetic matter from mother or donor to daughter or acceptor cells, e.g., along division of bacteria, single-celled fungi or somatic tissue cells of multicellular eukaryotes, encompassing both intracellular and extracellular mechanisms.

**Intergenerational inheritance:** Transfer of genetic and non-genetic matter from donor to acceptor organisms via asexual (budding) or sexual (oocytes, sperm) reproduction, which may also involve EVs (i.e., epididymosomes, prostasomes, uterosomes).

**Membrane-environment landscapes (MELs):** Configurations of membrane proteins in concert with PM lipids of specific composition and topography, which are transferred from donor to acceptor cells by EVs or micelle-like complexes.

**Micelle-like GPI-AP complexes:** Specific subtype of MELs consisting only of GPI-APs, (lyso)phospholipids and cholesterol, which on the basis of their amphiphilic character (self-)assemble (?) into micelle-like structures in order to ensure compatibility with the aqueous environment. Micelle-like GPI-AP complexes are released constitutively or in response to a specific stimulus (disease) and may be regarded as the most simple MELs. Their use for diagnostics (biomarkers) is currently being intensively investigated.

**Non-genetic matter:** Biological macromolecules and structures of specific composition and topography that are transferred via extra- and/or intracellular routes from donor cells or organisms to acceptor cells or organisms during intercellular or intergenerational inheritance and are capable of the replication and formation of a specific phenotype in the acceptor cells or organisms, but do not contain genetic matter, e.g., EVs, micelle-like complexes.

**Non-genetic inheritance:** Transfer of non-genetic matter from donor cells or organisms to acceptor cells or organisms and its replication and transformation into specific traits in the latter.

**SAW biosensing:** A method for the detection of the interaction of biological macromolecules assembled in a complex based on microfluidic chips and horizontal surface acoustic waves (SAWs). For this, the complexes become coupled via ionic and/or covalent bonds to the gold surface of the chips, into which tiny liquid channels have been integrated (Gronewold et al., 2005). For specific detection and characterization of the complexes, binding proteins or antibodies against components of the complexes are then injected into the chip channels, i.e., in the case of micelle-like GPI-AP complexes, antibodies against GPI-AP protein components, α-toxin which binds to the GPI glycan core or annexin-V which interacts with phosphatidylserine. The resulting mass increases on the gold surface lead to a shift to the right of the phase and/or a reduction in the amplitude of the SAWs, which propagate along the chip surface between fine electrodes. Such typical changes in the shape of the SAWs provide information about the presence in the sample, as well as the chemical composition and physical characteristics (viscoelasticity) of sample complexes, such as the micelle-like GPI-AP complexes.

**Science and technology studies:** Synthesis of heterogeneous trends of conceptions of the philosophy of science (including for instance “agential realism”), as well as the history and sociology of science, the aim of which is to describe the interaction (in complex networks or “apparatuses” of observation) of as many human and non-human–putatively previously unconsidered–actors as possible both in the production of scientific phenomena and in the assignment of meaning (“agency”), such as the differentiation between genetic and non-genetic matter of heredity.

**Self-assembly:** The spontaneous aggregation of all (newly synthesized) components present in a macromolecule to the structurally and functionally intact complex *de novo*, i.e., without the support of exogenous factors, such as binding, scaffolding or template proteins (for a review, see Hilander et al., 2021), which do not act as constituents of this complex. For example, the biogenesis of complexes consisting exclusively of nucleic acids and proteins, such as ribosomes (Pecoraro et al., 2021), signal recognition particles (for a review, see Massenet, 2019), bacteriophages, plant RNA viruses (e.g., tobacco mosaic virus), follows the principle of self-assembly, with its defined start and termination.

**Self-organization (self-templating, autopoiesis):** The incorporation of all newly synthesized components, which form a given complex, into pre-existing structures that contain the complete topographical information (for a critical discussion of the term “information” in life sciences, see Dickins, 2023) for the growth and replication of the structurally and functionally intact complex and involves the directional “organization” or “autopoiesis” at the level of subcellular and cellular systems, like a continuum without beginning and without ending. Thus, the biogenesis of complexes of proteins and lipids in general and components transferred by MELs to acceptor cells in particular, including GPI-APs and (lyso)phospholipids, follows the principle of self-organization, as well as mitochondria (for a review, see Grevel et al., 2019; Moulin et al., 2019), ER (endoplasmic reticulum) (Hsieh and Shan, 2021; Tirincsi et al., 2021; Lang et al., 2022; O’Keefe et al., 2022; Jung and Zimmermann, 2023), animal DNA viruses. Obviously, the constitution of macromolecules within biological membranes, with its defined topological orientation and determination of inside and outside, and the asymmetric distribution of its protein and lipid constituents between the inner and outer leaflets (Lorent et al., 2017; Lorent et al., 2020) makes the difference vs. self-assembly.

## Transfer of GPI-APs from micelle-like complexes to somatic cells

The method that can be used for the detection of transfer of micelle-like GPI-AP complexes and its phenotypic consequences is based on SAW biosensing. For instance, micelle-like GPI-AP complexes released by rat adipocytes were incubated with human erythroleukemia cells (ELCs) or rat adipocytes as acceptor cells lacking endogenous GPI-APs completely (as a consequence of defective GPI synthesis in mutant ELCs; see Hirose et al., 1992; Lazar et al., 1994) or partially (through inhibition of GPI synthesis by methyl-ß-cyclodextrin in adipocytes; see Ilangumaran and Hoessli, 1998). PMs were isolated and consecutively coupled by ionic and covalent bonds to the gold surface of the chips. The coupling resulted in a stable increase in the phase shift of the SAWs after washing of the chips. Incubation of the ELCs or adipocytes with isolated micelle-like GPI-AP complexes led to a time- and concentration-dependent increase in phase shift upon injection of antibodies against the GPI-APs CD55, tissue-nonspecific alkaline phosphatase (TNAP), CD73 (5′-nucleotidase) and acetylcholinesterase (AChE), reflecting their transfer from micelle-like complexes to the acceptor cells (Müller et al., 2021b). In contrast, the phase shift induced by antibodies against the transmembrane proteins band-3, glucose transporter-4 and insulin receptor did not increase, thus indicating their expression in ELCs and adipocytes, but failure to be transferred from the micelle-like complexes.

Interestingly, transfer of GPI-APs from complexes released by rat adipocytes to ELCs or to rat adipocytes was accompanied by significant stimulation of glycogen and lipid synthesis, respectively, in a concentration-dependent manner. These metabolic effects of the transfer of GPI-APs from micelle-like complexes could only be detected with acceptor cells with blocked or reduced expression of endogenous GPI-APs. Complexes reconstituted *in vitro* with purified GPI-APs CD73 and/or AChE supported their transfer to but were inactive in inducing metabolic effects in the acceptor cells.

## Transfer of GPI-APs between somatic cells

For demonstration of transfer of GPI-APs between somatic cells, differentiated human adipocytes as donor cells were grown in the upper compartment (insert well) of a transwell co-culture on the surface of a filter plate, which allows passage of large macromolecules and complexes, but prevents that of vesicles and cells. GPI-deficient ELCs (see above) were cultured in the lower compartment (bottom well) as acceptor cells. Analysis using SAW biosensing showed that GPI-APs are released during growth, differentiation and aging of the adipocytes and after passage across the filter plate transferred to the ELCs. Importantly, their number at the surface of the ELCs was positively correlated to stimulation of glycogen synthesis in the ELCs. Serum proteins and α-toxin (both via binding to the GPI anchor), bacterial PI-PLC (via cleavage of the GPI anchor) and antibodies against GPI-APs interfered with both transfer and upregulation of glycogen synthesis (Müller and Müller, 2022; Müller and Müller, 2023c).

These and a large number of additional experimental findings, e.g., co-culture of large mature and small young primary rat adipocytes (Müller and Müller, 2022; Müller and Müller, 2023c), suggest the possibility of a paracrine transfer of GPI-APs via micelle-like complexes between somatic cells of the same tissue depot with physiological consequences, e.g., from differentiated lipid-filled mature adipocytes to “empty” pre-adipocytes containing only a few small lipid droplets (LDs), to support energy storage. In contrast, the endocrine transfer of GPI-APs from peripheral tissues, e.g., adipose tissue, to distant tissue cells or blood cells with parallel stimulation of glycogen synthesis, as mimicked in co-culture with considerably efficacy, was found to be prevented by the presence of serum proteins in the top and bottom compartments (e.g., albumin, GPI-PLD) and thus is regarded as physiologically undesired.

## Transfer of GPI-APs to induced pluripotent stem cells (iPSCs)

The intercellular transfer of GPI-APs via micelle-like complexes was demonstrated not to be restricted to differentiated somatic cells (Müller and Müller, 2023c): Incubation of (commercially available) human iPSCs with micelle-like GPI-AP complexes released by rat adipocytes or with human adipocytes in co-culture and subsequent coupling of the PMs of the iPSCs to the SAW biosensing chip led to increases in phase shift upon injection of antibodies against the GPI-APs CD59, TNAP, CD73 and AChE, but not against CD55. These increases were dependent on the amount of complexes and the incubation period and thus reflect transfer of GPI-APs from the complexes or donor adipocytes to the acceptor iPSCs. In contrast, the phase shifts induced by anti-CD55 and anti-glucose transport-1 antibodies did not increase, which is compatible with expression of this GPI-AP and transmembrane protein, respectively, in iPSCs, but is not indicative of their transfer from adipocytes. Apparently, the transfer of GPI-APs is selective with regard to both the nature of the protein and the acceptor cell. Importantly, transfer of a (sub)set of adipocyte GPI-APs was accompanied by significant stimulation of lipid synthesis in the iPSCs. In contrast, complexes reconstituted *in vitro* only with AChE and/or CD73 or incubation without donor cells failed to affect lipid synthesis.

## A model for the intercellular transfer of GPI-APs

**FIGURE 2:**
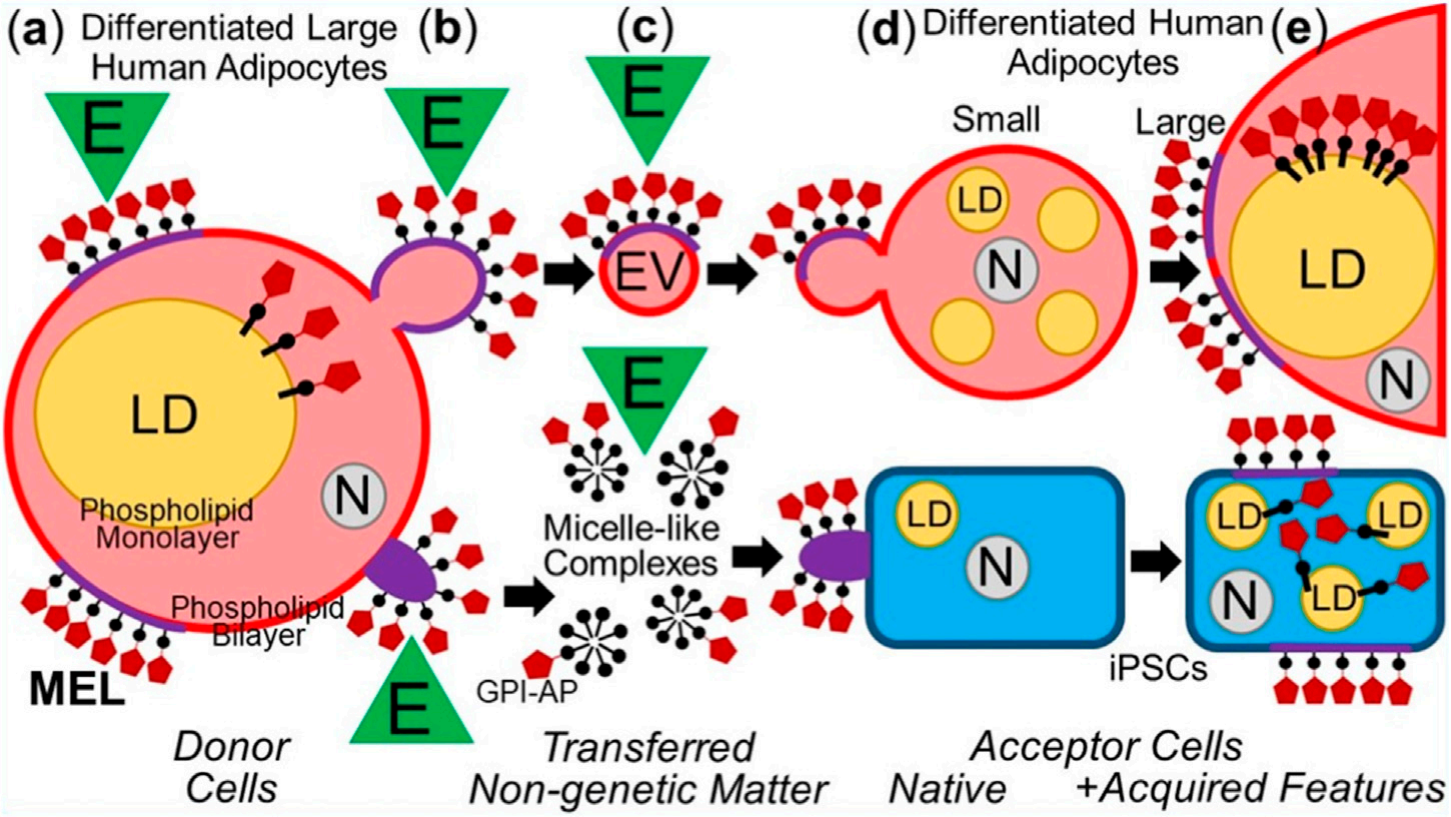
Model for the intercellular transfer of GPI-APs from somatic donor to differentiated or non-differentiated (iPSCs) somatic acceptor cells and physiological consequences. **(A)** Differentiated adipocytes express GPI-APs at the extracellular leaflet of PMs and are concentrated there at MELs consisting of (glyco-/sphingo-)phospholipids with long acyl chains, cholesterol, transmembrane proteins, peripheral proteins, intrinsically disordered proteins (Milles et al., 2018; for a review, see Uversky, 2013; Uversky, 2015) and cytoskeletal components (Sharonov et al., 2016). Environmental factors (E), such as mechanical forces, oxidative stress, ageing and nutrition, can affect the topography (composition and/or arrangement) of MELs (Levental et al., 2010; Lingwood and Simons, 2010; Levental et al., 2016). In addition, GPI-APs are located at the cytoplasmic face of lipid droplets (LDs) by insertion of their GPI anchor into the surrounding phospholipid monolayer, including 5′-nucleotidase CD73 and cAMP-binding ectoprotein/phosphodiesterase Gce1 (Müller et al., 1994; Müller et al., 2008b), which together contribute to the coordination of lipid synthesis and lipolysis (Müller et al., 2008d) by controlling the cAMP concentration at the immediate surface layer of the LDs (Müller et al., 2008c). **(B)** Mature lipid-filled donor adipocytes release MELs-expressing EVs or micelle-like complexes into the surrounding tissues which may be significantly stimulated by E. **(C)** During transfer of EVs and micelle-like complexes from donor to acceptor cells, the topography (composition and/or arrangement) of the MELs may be affected by E (e.g., blood pressure, radicals). **(D)** EVs and micelle-like complexes fuse with or insert into the PMs, respectively, of differentiated (e.g., small preadipocytes) or non-differentiated somatic cells (e.g., iPSCs). **(E)** In the case of adipocytes, the correct insertion of the MELs into and their redistribution from the PMs, in concert with subsequent translocation of the transferred GPI-APs to the surface of LDs leads to the upregulation of lipid synthesis and LD biogenesis (see **(A)**). N, nucleus.

In summary, the intercellular transfer of MELs from differentiated somatic donor cells to differentiated as well as undifferentiated somatic acceptor cells with the aid of EVs and micelle-like complexes can lead to altered (e.g., metabolic) phenotypes in the acceptor cells (Figure 2).

Specificity is guaranteed by various distinct mechanisms operating at the level of both acceptor and donor cells: With regard to acceptor cells, i) transfer of GPI-APs via EVs or micelle-like complexes seems to be restricted to very short distances, i.e., within the same tissue depot at a paracrine level, e.g., from large to small adipocytes at the immediate neighborhood (Müller, 2011), ii) endocrine transfer of GPI-APs via EVs or micelle-like complexes escaping into the circulation is apparently hampered by cleavage through GPI-PLD, a major component of mammalian serum, as well as the interaction with GPI anchor-binding proteins, among them as a minor component serum albumin, and certain GPI-PLCs, which become converted from a cleaving-into a binding-protein upon chelation of Ca^2+^/Mg^2+^ (Müller et al., 2019; Müller et al., 2021a), iii) the interactions prevalent during ii) are presumably modulated by a complex and competitive interplay between the phosphoinositolglycan portion of full-length GPI-APs which become released via EVs or micelle-like complexes, and phosphoinositolglycan moieties of anchor-less GPI-APs which are generated by certain GPI-PLCs in response to insulin or sulfonylureas (Müller et al., 2005; Müller and Müller et al., 2023c).

With regard to donor cells, i) the biophysical characteristics, such as their stiffness and viscoelasticity (Müller et al., 2020b), and ii) the velocity and pressure of the passing interstitial fluids or blood stream (Müller et al., 2020a) both determine the constitutive portion of the release of GPI-APs into EVs and micelle-like complexes (Müller et al., 2008a), and iii) may be further stimulated by exogenous signals through engagement of mechanisms, ultimately leading to certain alterations in biophysical and/or biochemical features of the donor membranes which facilitate their release, but still remain to be characterized in detail.

The findings of the local restriction of the effects of the transfer of subsets of GPI-APs via EVs or micelle-like complexes to the same tissue depots, i.e., at the paracrine level, are compatible with recent observations that the stimulation of lipid synthesis as well as glycogen synthesis are apparently complementary to the inhibition of the (isoproterenol-induced) lipolysis as well as (glucagon-induced) gluconeogenesis for transfer from large lipid-loaded adipocytes to small “empty” preadipocytes as well as glycogen-storing ELCs of high to those of low capacity, respectively (Müller and Müller, unpublished observations). It will be interesting to study whether the escape of subsets of GPI-APs from the tissue depots to blood or distant cells may exert unrelated (unwanted or even deleterious) effects in those cells which do not originally express them.

An interesting non-vesicular or non-micellar mechanism for the intercellular transfer of MELs seems to be represented by the direct transfer of (integral) membrane proteins of a specific area from the PMs of the donor cells to the PMs of the acceptor cells via direct cell-to-cell contact (Bouma et al., 1977; Huestis and Newton, 1986; Newton and Huestis, 1988). This process, described for the first time more than 40 years ago, and meanwhile called “trogocytosis”, is accompanied by the loss of MELs in parallel with specific functions in the donor cells and the gain of MELs in parallel with corresponding functions by the acceptor cells (Miyake and Karasuyama, 2021; Reed et al., 2021). This opens the provocative possibility that transfer, replication and phenotypic effect are not limited to certain subsets of GPI-APs but may be extended to specific membrane proteins, in general.

**FIGURE 3:**
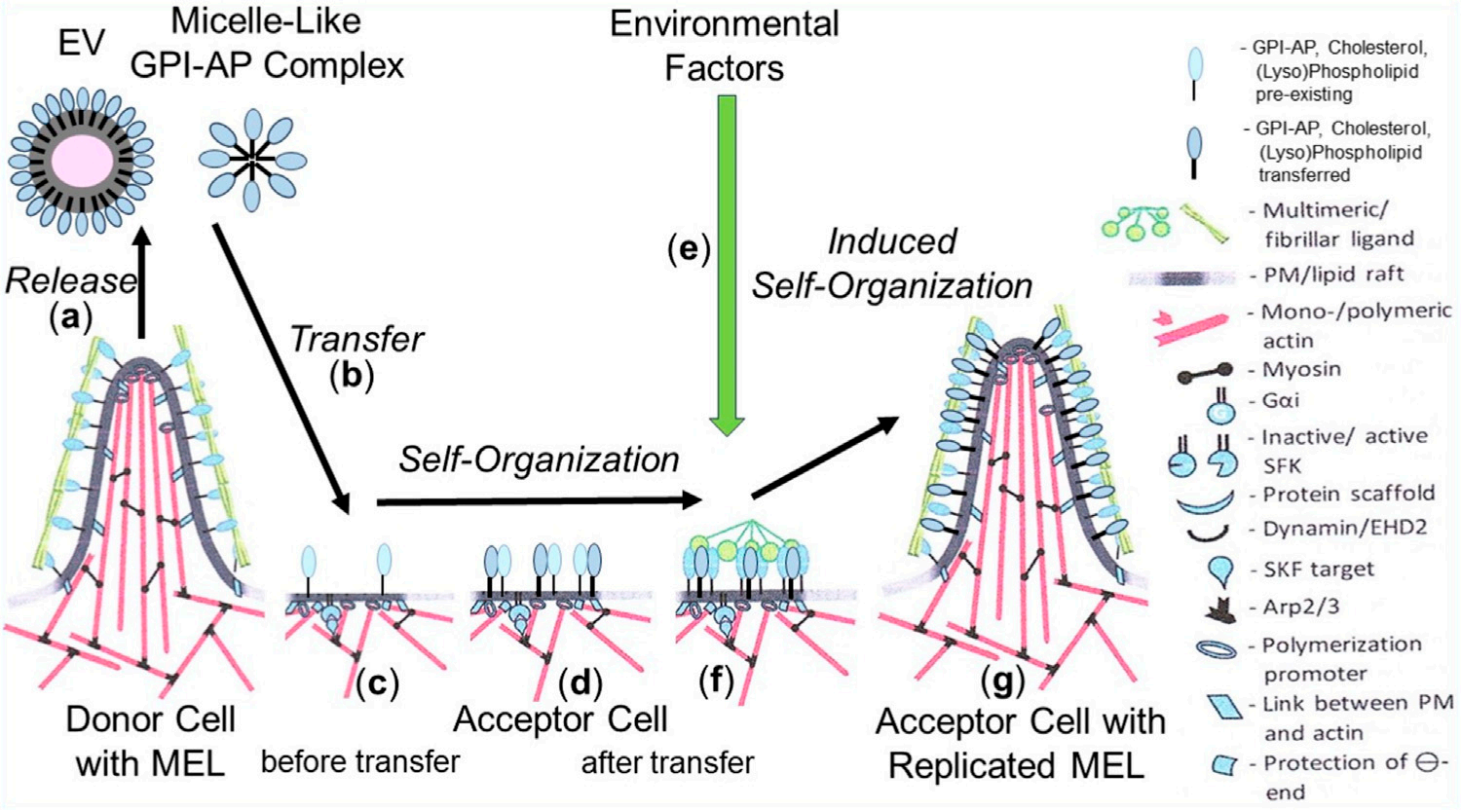
Model for the replication of MELs in acceptor cells by self-organization/self-templation upon their intercellular transfer and effect of environmental factors. **(A)** EVs and micelle-like GPI-AP complexes are released from MELs of donor cells. The former are then transferred to **(B)** and incorporated into **(C)** specific nano-/microdomains (pre-existing MELs) at the PMs of the acceptor cells equipped with all components organizing the directional “autopoiesis” at the level of subcellular systems for the growth and replication of the newly incorporated MELs **(D)**. **(G)** A complex molecular machinery operating at those sites and consisting of cytoplasmic and cytoskeletal components, including G proteins, dynamin, actin, myosin, Arp, is involved in the biogenesis of new MELs of the same specific topography at the PMs of acceptor cells as a mechanism of the growth and replication of MELs. **(E)** Environmental factors, among them stimuli leading to the formation of multimeric/fibrillar ligands **(F)**, in conjunction with mechanical forces (e.g., blood pressure) and/or biochemical factors (e.g., radicals, nutrients, chemicals) are required to induce the self-organization/self-templation of MELs with specific topography **(G)**.

Based on the available data, it is unlikely that transfer of a single GPI-AP or of the complete (sub)set of GPI-APs of the donor cells is sufficient or necessary, respectively, to induce phenotypic switches in the acceptor cells. Rather, it is reasonable to assume that only a few specific presumably functionally related GPI-APs together with certain (lyso)phospholipids and cholesterol manage to build up those nano- and microdomains of specific topography (Lingwood and Simons, 2010; Garcia-Parajo et al., 2014; Lorent et al., 2017; Mayor et al., 2023), among them certain protrusions, protuberances and blebs (Saha et al., 2016; Kalappurakkal et al., 2020; Saltukoglu et al., 2023). Those structures may be regarded as the smallest units of non-genetic matter of heredity, collectively referred to as MELs. MELs manage i) to be transferred to acceptor cells, ii) to switch the phenotype of acceptor cells, and iii) to be reliably replicated in acceptor cells. With regard to the involvement of PMs in determining the topography, orientation and inner/outer boundary of organisms, it seems likely that the molecular mechanism of replication of MELs follows the principle of self-organization or self-templating rather than self-assembly. Thus, the transferred GPI-APs, transmembrane and peripheral proteins in concert with (lyso)phospholipids and cholesterol become incorporated into already existing nano-/microdomains, which operate as pre-existing sites of “organization” and directional “autopoiesis” at the level of subcellular systems for the growth and replication of MELs (Figure 3).

**FIGURE 4:**
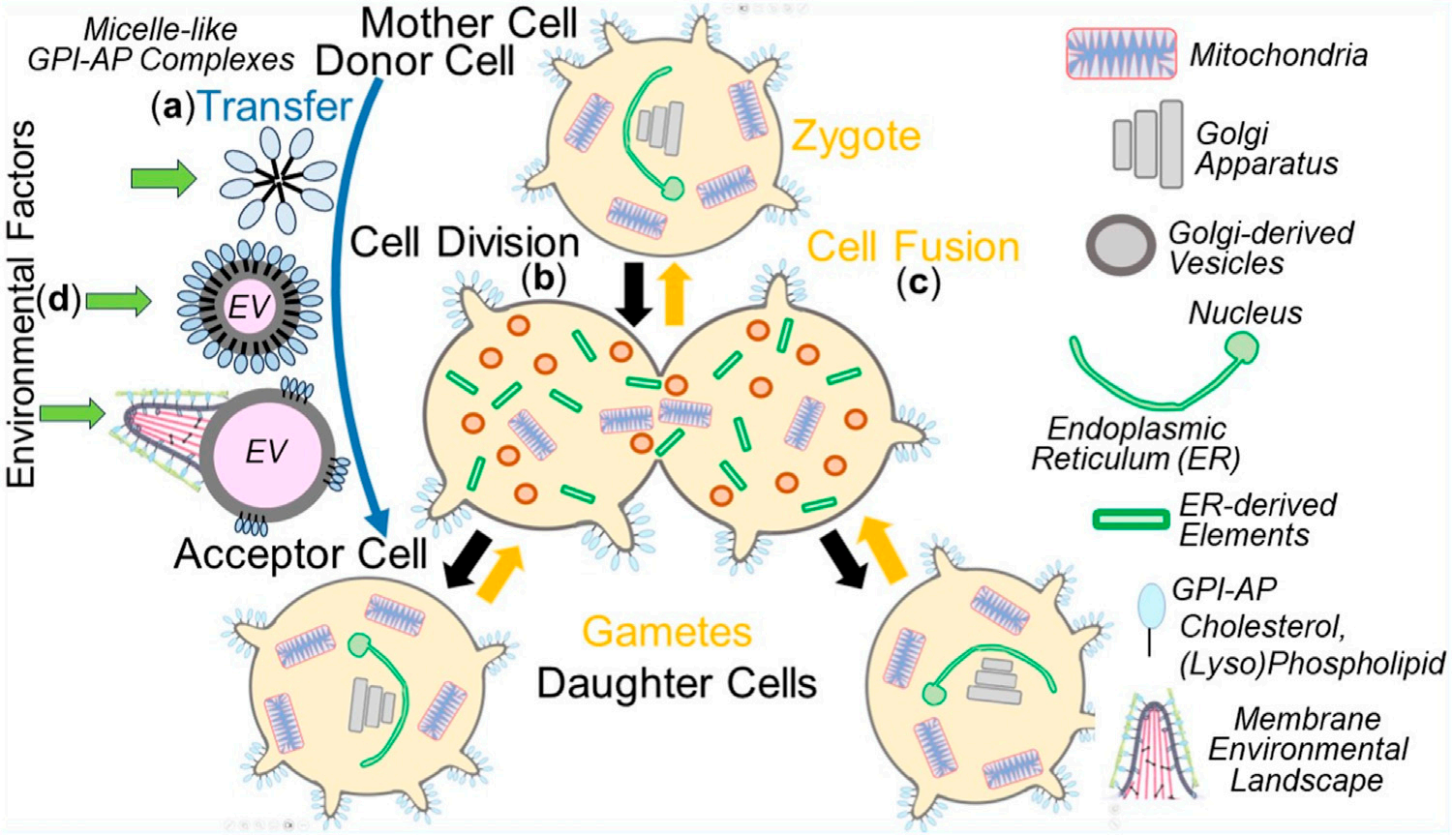
Extra- and intracellular inheritance of non-genetic matter between mother and daughter cells, gametes and zygotes, donor and acceptor cells, in the course of cell division, cell fusion or material transfer and effect of environmental factors. **(A)**, extracellular) Micelle-like GPI-AP complexes or native EVs induce the organization of new MELs in acceptor cells (see Figure 3) or EVs expressing MELs transfer them directly to acceptor cells (third option). **(B)**, cell division) PMs, including MELs, and organelles, such as mitochondria as well as Golgi apparatus and ER, fragmented into small Golgi-derived vesicles and ER-derived elements, respectively, in the mother cell are then distributed to the daughter cells during cell division. Those vesicles and elements then find each other in the daughter cells and fuse with each other to produce functional ER and Golgi apparatus. In conclusion, in daughter cells or zygotes, PMs, including the constituting MELs, mitochondria, ER, and Golgi apparatus, become replicated by self-organization/self-templation. **(C)**, cell fusion) PMs and organelles of the two gametes merge with one another in the zygote following cell fusion. **(D)** The susceptibility of the different non-genetic matter towards environmental factors during transfer is thought to vary considerably, with micelle-like complexes, native EVs and EVs expressing MELs being significantly more sensitive than PMs and organelles. The impact of structural and functional changes in PMs and organelles, which can be induced by environmental factors in the course of division of somatic cells and fusion of gametes, to the (patho-)physiology of daughter cells and zygotes, respectively, has not yet been adequately investigated.

As a consequence, the mechanism of copying non-genetic matter appears to follow the biogenesis of other cellular membranous structures, with each of them requiring complex molecular machineries, such as manifested in mitochondria (for a review, see Grevel et al., 2019; Pfanner et al., 2019) and the ER (Hsieh and Shan, 2021; Lang et al., 2022; Jung and Zimmermann, 2023). It is important to note that the intercellular transfer of non-genetic matter between donor and acceptor cells does not only take place using the extracellular route, i.e., via EVs and micelle-like complexes, but also engages intracellular routes, i.e., along cell division in the course of distribution of PMs and organelles between mother and daughter cells as well as gametes and zygotes (Figure 4).

## Societal and biological inheritance

We do not intend to present an–even short–outline of the cultural history of the thinking on biological inheritance in various political and societal systems over the centuries. This interesting topic has been addressed in depth by several very important and fascinating articles and books (Rheinberger and Müller-Wille, 2009; Szabo and Poczai, 2019; Poczai and Santiago-Blay, 2022). Nevertheless, some introductory remarks are presented, just to prepare the following considerations.

Until the end of the 18th century, there was no talk on biological “inheritance”, at least in terms of pure language use. Observations that have now been attributed to heredity as a matter of course–such as the fact that some physical or psychological peculiarities are limited to certain families, or the peculiar phenomenon that children sometimes look more like their grandparents in comparison to their parents–were already noticed in antiquity (Lesky, 1951). However, the terms “heredity” or “inheritance” owes its biological meaning, which is still dominant today, to the translation of their legal usage to phenomena related to the reproduction of organisms. Interestingly, before the end of the 18th century, as a rule, such a “transformation” simply did not happen. Of course, analogies between legal heirs and processes of biological inheritance became apparent with time, since the inheritance of goods and wealth is tightly linked to kinship and thus ultimately–from today’s perspective–to a biological relationship.

In the legal sense, inheritance refers to the distribution of goods and wealth in chronological order according to a system of rules and distinctions, that determines how goods and wealth should pass into the possession of other people after the death of their owner. The distinctions mostly concern the degrees of kinship, and the rules indicate which of these degrees of kinship are entitled to what share of the inheritance. Thus, in many regions of modern Europe, it was possible to establish rules of inheritance according to which only the first-born son was entitled to inherit, and indeed this held true for all future generations. At contrast, currently in many countries the law tells the society precise keys according to which at least part of the inheritance is to be divided equally among the children of next-generation. The historical and intercultural diversity of such legal regulations is beyond any suspicion. But the crucial point is that succession and succession rights are always subject to classifications and awarding rules.

These few fundamental considerations sufficiently exemplify what conditions had to be fulfilled before ideas of heredity could be transformed from the legal sphere to that of biological reproduction. The reproduction of organisms had to be able to appear as a process in which something was passed on at all and distributed according to applicable rules. However, it is precisely this perspective that is missing in the work of great pre-modern natural philosophers such as Aristotle, William Harvey or René Descartes. Speculations and unsystematic observations about the act of procreation, as found in their published work, hint to other directions and connections. Most importantly, genealogies had to be traced, distinctions introduced, links between acts of procreation established, until finally the outlines of a structure became recognizable, to which the meaning and agency of “heredity” could be critically and productively applied. To understand reproduction as heredity presupposes that human and non-human (f)actors are at work in the procreation of organisms that persist beyond the mere moment of procreation.

## Intergenerational inheritance of non-genetic matter through “Gemmules”

From the midst to the end of the 19th century, the identification of the material basis of the transfer of specific features from parents to offspring received increasing interest from several and at those times still converging areas of biology, such as morphology (advances in microscopy), developmental cell biology (nucleus manipulation) and botany (hybridization experiments). In fact, each of these driving forces gained further impact soon after the first publication of Charles Darwin’s On The Origin of Species in 1859 in the course of the debate on the mechanisms of biological evolution and, in concert, of the variation between individual organisms (see Dunn, 1965, p. 34; Gasking, 1967, p. 161; Geison, 1969, pp. 375, 385-386; Robinson, 1979, p. xiii; Cowan, 1985, Ch. 5; Olby, 1985, Ch. 3; Bowler, 1989, p. 46; Gayon, 1998, Ch. 1). Certainly, in earlier periods the operation of some materials, particles, corpuscles of inheritance etc. had been postulated by famous authors, among them Buffon, Diderot and Maupertuis. However, the origin of the conception of living matter, discrete units or material building blocks as the basis or substrate of heredity, which act in a continuous “line” with our own development, may be found at considerably later times in the “Physiological Units” as defined by Herbert Spencer (1864) in his Principles Of Biology or in the “Gemmules” as introduced by Charles Darwin (1868) in his The Variation Of Animals And Plants Under Domestication. Importantly, the conceptions of both Spencer and Darwin must not be misinterpreted in terms of “particulate” heredity, as sometimes used to characterize the mechanism or material basis of the transfer of specific features in discrete units from parents to offspring, from one generation to the next, without “blending”, i.e., amalgating or mixing of the various features of the parents to yield novel phenotypic combinations in the offspring. No doubt, Spencer and Darwin supported the “blending” rather than the “particulate” version of the material or particle conception of inheritance. Albeit Spencer’s conception was published 4 years earlier than Darwin’s one, presumably due to the more concrete and mechanistic understanding of the former, Darwin’s conception gained much greater attention and impact for the debates about variation, inheritance, development and generation during the following decades, in both confirming and disproving or even abusing fashion (see Robinson, 1979, p. 24; Churchill, 1987, p. 343; Endersby, 2003, p. 81).

It is not marginal that Darwin did not agree with the canonical conception of variation and heredity as mutual complementary consequences emerging out of a single process and connected by a single efficient cause. At variance, he emphasized that variation and heredity represent antagonistic actions opposing each other (Bowler, 1989, p. 25; Hodge, 1989, p. 277; Gayon, 1998, Ch. 1). Darwin interpreted variations between parents and offspring as incidents occurring by chance, emerging out almost exclusively by altered conditions of life, and operating against a vast number of inherited features. Thus, for Darwin inheritance was the rule and non-inheritance, i.e., variation, the exception. And for him inheritance seemed to be guaranteed by reliably functioning of the reproduction systems which transfer the features in authentic fashion from parents to offspring.

The central conception underlying Darwin’s theory of inheritance (and growth, development, reproduction and repair) or “Pangenesis” theory and its explanatory potential for the well-known observation that a character possessed by some remote ancestor, but not expressed in the parents, suddenly reappear in the offspring, is that ‘an organism does not generate its kind as a whole but each separate unit generates its kind’ (Darwin, 1871), i.e., ‘every separate part of the whole organization reproduces itself. So that ovules, spermatozoa, and pollen-grains, - the fertilized egg and seed, as well as buds, - include and consist of a multitude of germs thrown off from each separate part or unit’. Certainly, one of the most curious phenomena for Darwin was this phenomenon of intermittent characters, who disappear once in the offspring and reappear in the next-generation, as he stated already in the first chapter of On the Origin of Species (Darwin, 1859, p. 32). What caught Darwin’s attention was the “life of their own” of qualities, the distribution of which among the offspring was apparently not explainable by external circumstances but had to be traced back to a hidden mechanism. The epistemic space in which such peculiar characters circulated could no longer be confined to the individual relationship between parental progenitors and their offspring.

**FIGURE 5:**
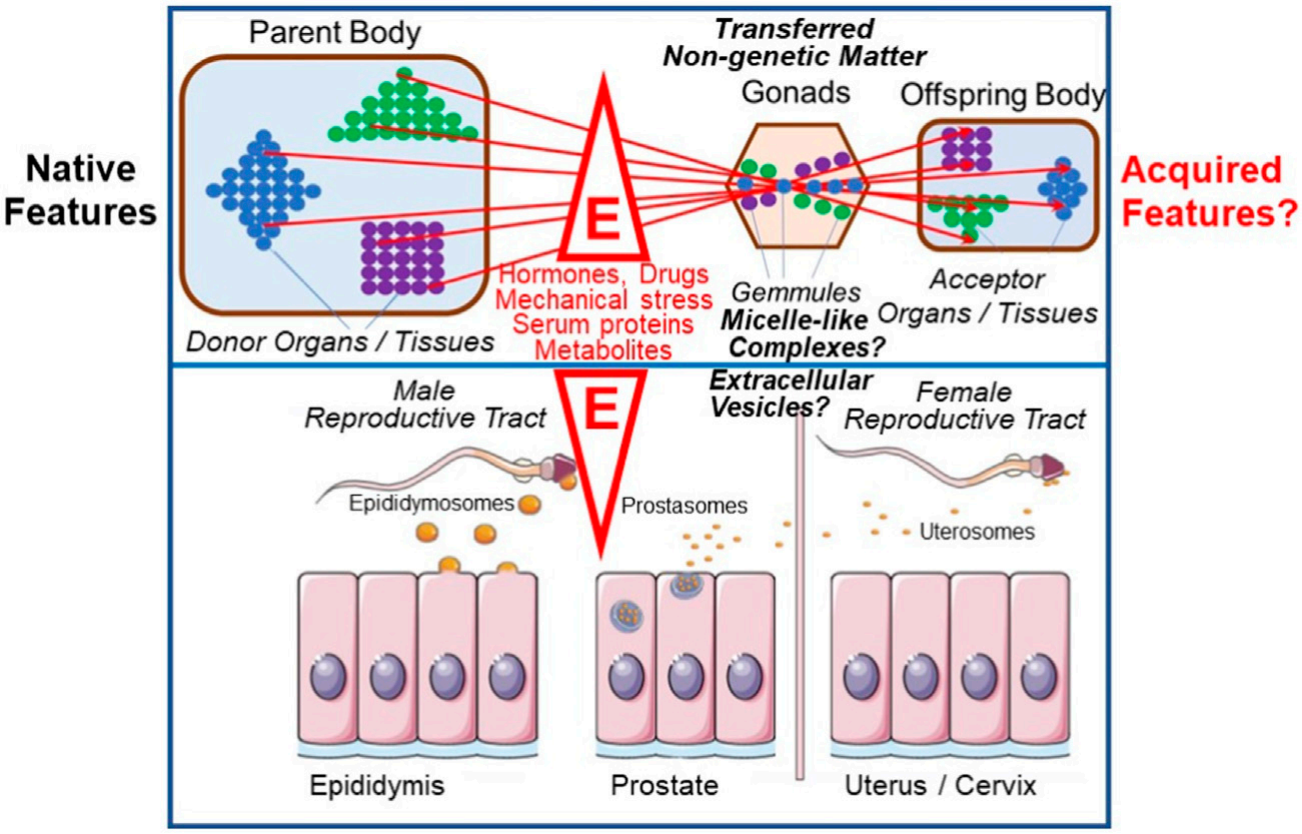
Intergenerational inheritance of (acquired) traits and possible revival of Darwin’s “Gemmules” and “Pangenesis” theory (for details, see text). E, environmental factors; colored circles, “Gemmules” of different origin and fate; triangle, square and diamond, cells, organs and tissues releasing “Gemmules”.

Darwin’s two-volume work on The Variation Of Animals And Plants Under Domestication was initially intended to form a part of On The Origin Of Species, but occupied the author for nearly a decade longer. Here he collected all that he had been able to find about variations and their transmission among breeders as well as in the medical and natural history literature. In chapter 27 he tried to make a ‘plausible connection’ between the most important observations to him: sexual reproduction, grafted hybrids, xenia, development, the functional independence of the particles, elements or units of the body, as well as variability and heredity. He found this connection in his ‘provisional hypothesis of Pangenesis’ (Darwin, 1868, p. 374). At a first glance surprising, Darwin firmly believed in Lamarck’s theory of the inheritance of acquired traits and proposed the existence of so-called “Gemmules” as the underlying mechanism: He defined them as small particles which become released by each cell, organ or tissue of the donor organism or parental body, and then transported via body fluids to the gonads to accumulate there, e.g., in the neighborhood of egg cells. After transfer to the acceptor organism, e.g., the offspring body, the “Gemmules” reassociate to the same type of cell, tissue or organ from which they had been originally released, driven by some ill-defined forces (Darwin, 1869). Their characterization as a curious intermediary mechanism of biogenesis encompassing elements of both self-organization/autopoiesis and self-assembly/*de novo* synthesis deserves more detailed analysis in the future (Figure 5).

Thus, according to Darwin’s “Pangenesis” theory, not only were all the relevant characters of an organism gathered and assembled together as particles or germs in the sex cells, but also countless particles or germs came from more distant ancestors and could be passed on through generations in a dormant state before being re-formed, by whatever circumstance. For Darwin, this solved one of the most pressing problems of heredity, which bothered breeders as “distant reversion” or “atavism” and to which he returned again and again. Like the spontaneous and unpredictable occurrence of variations, for Darwin they were indications of autonomy, irregularity, and chance, that characterize life and its development (Voss, 2007). The stronger and weaker characteristics of features could also be attributed to different quantities or more or less pronounced penetrating power of the “Gemmules”. For Darwin, the problem of development was also traced back to a material substrate of heredity. It is a characteristic of the theories of heredity of the second half of the 19th century that, although they increasingly separated the phenomena of heredity and development, they still tried to explain them uniformly and jointly.

Is it possible that the extracellular transfer of non-genetic matter via EVs and micelle-like complexes, as had been previously demonstrated between somatic (differentiated and non-differentiated) donor and acceptor cells with phenotypic effects on the latter, also takes place between organisms? The first hints for this provocative view were provided by studies with EVs of donor and acceptor cells of the mammalian reproductive tract (Rooney et al., 1996; Babiker et al., 2002; 2005; Ekdahl et al., 2006; Griffiths et al., 2008a and b; Vickram et al., 2020; for a review, see Kirchhoff et al., 1997; Spadafora, 2017). In some cases, phenotypic effects have also been reported in acceptor animals (for a review, see Yanez-Mo et al., 2015). If the new phenotype also appeared in the next-generation, one may indeed argue for an intergenerational sexual inheritance of relevant non-genetic matter. And this could actually be interpreted in the sense of Darwin’s “Pangenesis” theory (Darwin, 1871). Remarkably, Spadafora and coworkers have recently found that DNA- and RNA-containing exosomes are released into the circulation and sometimes fuse with epididymal spermatocytes in various experimental systems (for a review, see Spadafora, 1998; Smith and Spadafora, 2005). This has been interpreted in the sense of a transgenerational flow of extrachromosomal RNA linking somatic and germ-line cells and, ultimately, of the formation of next-generation organisms (Vitullo et al., 2012; Cossetti et al., 2014). Spermatozoa are thought to act as bridging agents in this process by gathering and assembling the somatic materials and transferring them to the next-generation (for a review, see Spadafora, 2008). Spadafora (2017) concluded that ‘on the whole, this phenomenon is compatible with a Lamarckian-type view and closely resembles Darwinian pangenesis.’

## Intergenerational inheritance of non-genetic matter through “Stirps”

Darwin’s cousin Francis Galton, a polymath who made important contributions in many fields, including meteorology, statistics, psychology, biology (heredity, eugenics), and criminology, is considered by some historians of science to be the actual founder of modern heredity research (Olby, 1985, p. 55-63; Gayon, 1998, pp. 105–106). One does not have to share this view, but it is certain that Galton was one of the first who focused on heredity as the center of theoretical considerations about the phenomenon of life and developed a rich arsenal of analogies and figures.

In his essay A Theory Of Heredity, Francis Galton (1876, p. 330) repeatedly expressed the same idea as Darwin had done a few years earlier, with emphasis on the following important differences. His theory is based on four postulates: i) For every independent “unit” of a body (i.e., cell, tissue, organ), a germ is responsible. ii) The sum of the germs that make up the “Stirps” (Latin for “sprout” or “tribe” in the sense of family line, head of the family) in the fertilized egg is much greater than the sum of the germs that develop in each individual. iii) The non-developing latent germs form a ‘residue’, the most vital half of which enters the germ cells of the organism. iv) Specific forces of attraction and repulsion of the germs guarantee their regulated structural formation and individual development, a superordinate vital force seems to be dispensable (Galton, 1876, p. 331), a view shared by Darwin. In summary, heredity can only be explained if one assumes an organic structure that lasts through generations and carries or determines them to a certain extent. The “Stirp” is the sum of all germs, particles or “Gemmules” that, according to all theories of organic units, become deposited in the freshly fertilized egg cell. In fact, for Galton, heredity is determined by “Stirps”, which includes both genealogical and cytological relationships, insofar as the former must somehow be represented in the latter.

The ‘sexual elements’, Galton stated, ‘I see directly derived from the residue and do not claim’—in contrast to Darwin—‘that the germs can migrate freely’ (Galton, 1876, p. 343). In this way, Galton concluded that ‘direct descent’ in the sense of direct parenthood, i.e., procreation in the pre-modern sense, ‘is completely untenable in the common meaning of this expression’. According to Galton (1872, p. 346) in his treatise On Blood Relationship, it is precisely this everyday meaning of descent that is responsible for the fact that the actual phenomena of heredity often seem so ‘capricious’ to us, a curiosity often termed “distant reversion”. Thus, the actual hereditary relationship does not connect the parents with the offspring, but the primary elements of the two, as they exist in the newly fertilized egg. It will therefore only unnecessarily increase our ignorance ‘to treat heredity facts at the level of ordinary lines of descents–that is, from the persons of the parents to those of their children’ (Galton, 1872, p. 401).

Everything that reaches the descendants from their ancestors must have been packaged into “Stirps” released from the latter (Galton, 1876, p. 331). Consequently, heredity during Galton’s time can be characterized more accurately as an “epistemic space”, in contrast to other objects of biological research, which were defined as “epistemic things” in the sense of other experimental systems (Rheinberger, 2006). In On Blood Relationship, Galton (1872) distinguishes between “patent” and “latent” elements, which are not to be confused with the relationship between dominant and recessive hereditary factors as defined later. However, these probably mark out the epistemic space that has become constitutive for the development of classical genetics. As with Darwin, it was the curiosity of “distant reversion” that led to a long-term decisive discrimination in its comparative marginality and unpredictability for the inheritance process, namely, that between the transmission of hereditary elements on the one hand and their role in the development of the organism on the other.

## “Gemmules”, “Stirps”, GPI-APs, EVs, and the inheritance of acquired traits

As already mentioned, Francis Galton’s considerations led from a physiological to a statistical theory of heredity and finally to the postulation of the law of ancestral heredity. Importantly, only a few years earlier he had demonstrated in extensive infusion and cross-circulation (parabiosis) experiments with rabbits that Darwin’s speculation of a systemic circulation of “Gemmules” was definitely false, at least with blood as the “transport” medium. But rather than completely declining “Pangenesis” theory, Galton tried his best to rescue some parts of it, possibly to avoid withdrawal of his “Stirps” theory as the final outcome. Eventually, as a consequence, Galton (1865, p. 322) hypothesized that human beings are ‘passive transmitters of a nature that has come to us and that we do not have the capacity to change’. Therein lies the central point of his concept of heredity. Jean Gayon (1998, p. 9) has pointed out that Darwin sometimes referred to his theory of ‘descent with modification’ as a theory of ‘inheritance with modification’. This emphasis on “modification” has not been adopted by Galton (1865, p. 324).

Like Darwin, Francis Galton related the heredity process to a system of circulating germs, but according to his conception these are not derived directly from the body of the parents. Rather, ancestors and descendants are linked through a “line” of germs in which the inheritance process takes place. Therefore Dalton (1876) first proceeded from the assumption, shared with Darwin, that organic bodies, i.e., cells, tissues, organs, harbor–unspecified—‘organic entities’ that possess special properties and are to a certain extent independent of each other. However, he then divided these entities or units into “innate” and “acquired”. The former alone are decisive for his theory of heredity. Darwin’s “Pangenesis” hypothesis, which was also a convenient means of explaining the inheritance of somatically acquired traits, was thereby firmly rejected by Galton.

Most relevant, Francis Galton shared the opinion of Darwin what could be termed a “maturational” rather than a “directional” mode of heredity. According to the “maturational” conception, the consequences of inherited germs for the offspring are due to the direct action of the germs themselves rather than indirectly through “mere” initiating, induction, guiding or directing of their growth and development to the corresponding bodily constituents, i.e., cells, tissues and organs (as assumed by the “directional” conception). The “maturational” conception relies on one of Galton’s hypotheses that each of those quasi ‘independent bodily units’ must have emerged from a distinct germ. Thereby Galton neglected the possibility of the production of the organismal features by the germinal units without the transfer of specific isolatable and discrete materials from one generation to the next, which finally constitute the individual’s tissues, organs and body. Thereby he ignored the possibility of the generation of action of the bodily constituents other than as a consequence of a one-to-one correspondence between the germinal particles and the organismal units, such as cells, tissues, organs or phenotypic traits. Another hypothesis of Galton reflected this “maturational” conception, in that the assembly of those bodily units is driven solely by the mutual affinities and repulsions of the distinct germinal particles, which thereby ultimately constitute the total physical and spatial structure and organization of the emerging body.

In fact, the “directional” conception of heredity is not compatible with a number of Galton’s assumptions, i) the one-to-one correspondence between distinct “Gemmules” and each physical constituent of the organism, and ii) the generation of the structural organization of a body by the corresponding organization already inherent in the germinal matter, i.e., “only” multiplying and growing to the adult organism with the aid of some ill-defined forces acting between them. Apparently both Galton and Darwin were unable to consider an alternative to the “maturational” conception of inheritance. By nature, a “maturational” conception of inheritance *per se* seems to be more compatible with the inheritance of acquired traits compared to a “directional” one, the view shared by Darwin rather than by Galton.

In addition, Galton strongly believed on the “invariant” rather than “contextual” mode of inheritance. Accordingly, each active “Gemmule” or element specifically contributes to the next generational cell, tissue or organ inertly and completely independently of the other units or elements transferred together with it as well as of environment factors or the context in which it happens. At variance, the “contextual” mode considers the putative differential effects of the (individual) background, in the broadest sense, on the activity of the inherited “Gemmules” or elements which is constituted by the neighboring particles as well as the surroundings of the particles. On the basis of the neglection of the “contextual” mode, Galton insisted on the assumption that the “distant reversion” of organisms to features which are not expressed in the parents requires the production of a multitude of “Gemmules” within a given “Stirps” in the parents, many of which are inactive, undeveloped or latent, but retain their developmental capacity and vitality following their transfer to the offspring. This presumably represents the ultimate reason why Galton created the term “Stirps” for the total of the majority of inactive dormant particles and the minority of active developed particles.

Certainly, Galton’s explanation of the phenomenon of “distant reversion” with undeveloped or latent germinal matter critically depends on the “invariant” conception, i.e., the postulate that the inherited “Gemmules” exert their effect on the development of cells, tissues and organs of the offspring body in exactly the same fashion as they already did in the ancestor body. Interestingly, the “contextual” mode of Galton’s inheritance of “Stirps” is fully compatible with “distant reversion”, taking into account the transient recurrence of the corresponding intrinsic or extrinsic causal conditions, which were responsible for the production of a specific feature out of a specific “Gemmule” in an ancestor. No doubt, this mode of thinking is typical for modern molecular biology in that phenotypic plasticity, i.e., differential transformation of a given gene into distinct phenotypes, is regarded to be caused by the context in the broadest sense, i.e., by the interaction with other genes and the environment. Interestingly, even the Mendelian laws of dominant and recessive inheritance may be interpreted in terms of interactions between alleles and do not obey the principles of dormancy, inactivity, latency, or some inactivation mechanisms of genes. Even the action of regulatory genes does not fit to the “invariant” mode since their action seems to rely on the interaction between inherited “Gemmules”, i.e., between regulatory and regulated genes, rather than on the differential development between those. In fact, the operation of regulatory genes represents one of the mechanisms through which inherited “Gemmules” are prevented from causing the same effects as they did in the ancestor without being transferred in a dormant, inactive, undeveloped or latent state. In conclusion, Galton’s failure to consider alternatives to the “invariant” and “maturational” conceptions of inheritance seems to explain his rather skeptical to up to neglecting attitudes towards the possibility of the inheritance of acquired traits. In fact, a “conceptual” and “directional” conception could explain the transfer of characteristics adapted by the parents in response to specific environmental conditions and persisting in the offspring despite absence of those conditions in their life world.

## Micelle-like GPI-AP complexes–biochemical experiments hinting to their transfer, replication and induction of (metabolic) phenotypes

The conception of the inheritance of metabolic phenotypes, such as upregulation of lipid and glycogen synthesis, with MELs transferred via EVs or micelle-like GPI-AP complexes representing some of the relevant materials, as outline above, is compatible with the “contextual” mode of inheritance: Environmental factors and conditions of life, e.g., mechanical and oxidative stress, nutrition, neighboring cells, cell density, prevalent in the parental organism, may alter the topography (composition, arrangement) of some of their MELs (e.g., blebs, protuberances, protrusions, invaginations), leading to specific distortions of the PM “shape” or “form” (Jacobson et al., 2019; Kalappurakkal et al., 2020; Mayor et al., 2023; Saltukoglu et al., 2023), which become transferred to and copied by the offspring organisms.

**FIGURE 6:**
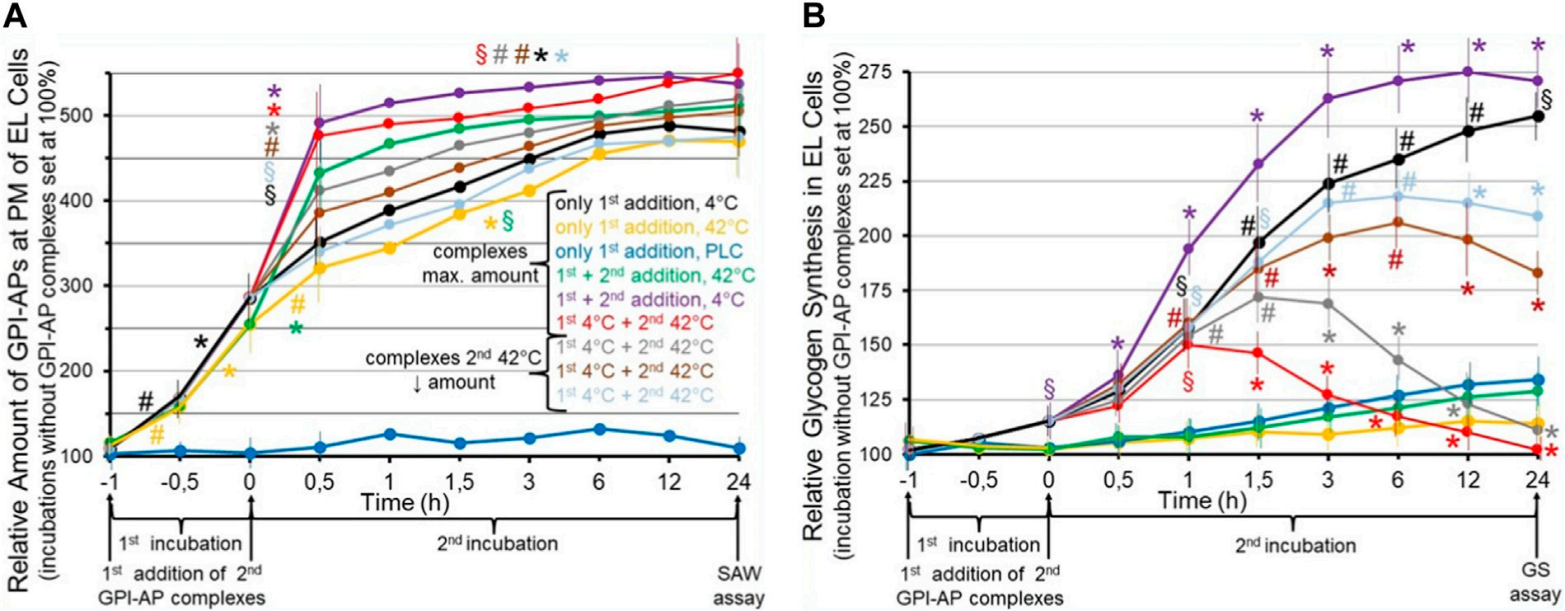
Effect of the transfer of normal and environmentally induced alterations of micelle-like GPI-AP complexes on the metabolic phenotype of the acceptor cells. Cultured GPI-deficient erythroleukemia (EL) cells (Hirose et al., 1992; Lazar et al., 1994) were incubated in the absence (control set at 100%) or presence of micelle-like GPI-AP complexes reconstituted from total full-length GPI-APs, which had been prepared from primary rat adipocytes, cholesterol and (lyso)phospholipids (Müller et al., 2020a; Müller et al., 2021b) and then left for 6 h at 4°C (black line) or 42°C (orange line) or treated with bacterial phosphatidylinositol-specific phospholipase C (PLC, dark-blue line) for enzymic removal of the GPI-AP protein moieties from the complexes (Davitz et al., 1986; Wong and Low, 1994) for 1 h at 37°C (first incubation from −1 to 0 h). Thereafter, portions of the cells (at identical number) were continued to be incubated for 24 h (second incubation from 0 to 24 h) without a second addition of complexes (black, orange, dark-blue lines) or after a second addition of micelle-like GPI-AP complexes at maximal amount (green, pink, red lines) or at decreasing amounts (1:3, grey line; 1:10, brown line; 1:30, light-blue line) which had been pretreated at 42°C (green, red, grey, brown, light-blue lines) or at 4°C (pink line) for 6 h. After termination of the second incubations at time point 24 h, the cultured EL cells were divided into two portions each and then assayed for **(A)** the relative amount of full-length GPI-APs expressed at the PMs of the EL cells by SAW biosensing (Gronewold et al., 2005; Andrä et al., 2008) and **(B)** relative glycogen synthesis in the EL cells using radiolabeled glucose at 2 mM (three to five independent cell cultures with incubations in quadruplicate using two different preparations of complexes and assays in triplicate, each; mean ± S.D.; *p ≤ 0.01, ^#^ *p* ≤ 0.02, ^§^ *p* ≤ 0.05) as outlined in detail previously (Müller et al., 1997). **(A)** The SAW biosensor monitors the transfer of full-length GPI-APs to the PMs of EL acceptor cells in course of the incubation with the micelle-like complexes harboring GPI-APs (compared to complexes lacking them; dark-blue lines). Significant differences vs incubation under identical conditions in the absence of complexes are given for the transfer of GPI-APs separately for both the first (from −1 to −0.5, −0.5 to 0 h) and second (0–0.5, 0.5–24 h) incubation at the various conditions each. **(B)** Glycogen synthesis is measured as a consequence of the transfer of full-length GPI-APs to the EL cells (compared to effects in response to incubation with complexes lacking GPI-APs; dark-blue lines). Significant differences between the first and second incubation at 42°C (pink line) and the various other incubations are given for each time point. Furthermore, for the first and second incubation at 4°C significant differences vs incubation in the absence of complexes are given for each time point.

It is of crucial importance that MELs are susceptible to environmental factors and manage to respond with specific changes in their topography, assembly state and three-dimensional configuration. Those changes may affect a specific function or the complete phenotype of a given cell. And these environmentally induced changes can be replicated by the incorporation of newly synthesized protein components into the altered MELs. This results in adaptation of the specifically altered topography by the newly replicated MELs which will be consequently termed “membrane environment landscapes” or MELs, just to stress the tight interaction of membranes, environment and PM “shape” or “form” (“landscape”). Taken together, the replication and transfer of MELs and their environmentally induced topographical alterations, which may be regarded as “non-genetic mutations”, could therefore represent a mechanism for the inheritance of acquired traits. The following experimental set-up provides first hints for the demonstration of environment-induced alterations of MELs and their intercellular transfer (Figure 6).

These results suggested that incubation of GPI-deficient EL cells with micelle-like GPI-AP complexes pretreated at 4°C (first addition) causes significant increases in the amounts of total rat adipocyte GPI-APs which can be recovered from their PMs in time-dependent fashion (Figure 6A, black line). The specificity of transfer of GPI-APs to the acceptor cells was confirmed by incubation with complexes harboring lipolytically cleaved GPI-APs which completely prevented increase of GPI-AP expression at the EL cells (dark-blue line). Pretreatment of the complexes at 42°C did not significantly alter the efficacy of transfer (orange lines). Transfer of GPI-APs was further stimulated in course of a second addition of complexes after 1 h, irrespective of whether being treated at 4°C (pink line) or 42°C (green line). Transfer was strictly dependent on the amount of complexes as shown for the second addition (red, grey, brown, light-blue in that ranking order of decreasing amount and transfer). Thus, the experimental set-up enabled monitoring of the transfer of full-length GPI-APs to GPI-deficient acceptor cells and did not reveal any difference in the transfer efficacy between complexes pretreated at 4°C and 42°C.

Strikingly, transfer of total rat adipocyte full-length GPI-APs to GPI-deficient EL cells upon the first addition of complexes pretreated at 4°C was found to be accompanied by significant stimulation of glycogen synthesis following the first and second incubation in time-dependent fashion (Figure 6B, black line). As expected, complexes lacking GPI-APs did not significantly upregulate glycogen synthesis (dark-blue line), and complexes pretreated at 42°C completely failed to promote glycogen synthesis, presumably due to heat-induced conformational changes of relevant GPI-AP protein moieties (orange line). This was confirmed by a second addition of micelle-like GPI-AP complexes pretreated at 42°C (green line) after 1 h which had no effect during the second incubation, whereas complexes pretreated at 4°C provoked a significant additional enhancement of glycogen synthesis in time-dependent fashion (black line). Strikingly, a second addition of complexes pretreated at 42°C to EL cells which had already been incubated with complexes treated at 4°C caused significant declines in glycogen synthesis in course of the second incubation compared to complexes pretreated at 4°C (Figure 6, pink line), with the maximal amount being most efficient (red line), followed by 1:3 (grey line), 1:10 (brown line) and 1:30 (light-blue line) dilutions, in that order of reduced inhibition of the glycogen synthesis left.

In extension of previous findings (Müller and Müller, 2022; Müller and Müller, 2023c), it has been concluded that transfer of full-length GPI-APs from micelle-like complexes to GPI-deficient acceptor cells leads to a change in their metabolic phenotype, here upregulation of glycogen synthesis, which is maintained for 2 weeks at least, i.e., in the course of several passages of the cultured EL cells. This led to the speculation that micelle-like GPI-AP complexes act as a matter of biological inheritance upon their transfer from donor to acceptor (somatic and possibly also germ-line) cells. In this experiment, the propagation, and the effect of an environment- (temperature-) induced–and presumably structural–alteration of the GPI-APs, which apparently is fully compatible with their transfer to, but interferes with the accompanying switching of the metabolic phenotype in the acceptor cells with already transferred functional GPI-APs, was studied. Interestingly, transfer of the altered GPI-APs to acceptor cells which had already received functional GPI-APs of the same type during a preceding transfer caused the concentration-dependent antagonism of this phenotype to up to its complete blockade.

Thus, the–presumably structural–alteration of the GPI-APs seems to propagate to normal GPI-APs which are thereby converted to non-functional ones and consequently fail to switch the metabolic phenotype. The mechanism underlying this propagation may rely on the direct physical contact of the GPI-APs following their lateral movement along the outer leaflet of the PM bilayer. This would explain the rather slow kinetics of the antagonism compared to a direct inhibitory effect (e.g., key-and-lock) and may be reminiscent to the replication of “protein genes”, such as prions. In any case, full-length GPI-APs, embedded in MELs together with transmembrane proteins, peripheral membrane proteins, cytoskeletal elements, cholesterol and (lyso)phospholipids, and displaying a specific topography, become transferred from donor to acceptor cells. Following their arrival, they manage to replicate their structural and functional changes, as provoked by environmental factors, in course of “self-organization” or “self-templating” or “autopoiesis” onto their pre-existing counterparts in the acceptor cells using a non-genetic mechanism. Consequently, MELs in general, and GPI-APs in particular, seem to be prone to act as non-genetic matter of biological inheritance which displays exquisite responsiveness towards environmental factors and thereby could provide a mechanistic basis for the inheritance of acquired traits. The underlying mechanisms of self-organization or self-templation or autopoiesis of MELs may lead to causal induction of the same specific topographical (MELs) and phenotypic (metabolic) features in the descendants as those acquired by the ancestors, i.e., mediate causal specificity (for a discussion of this term, see Vecchi et al., 2019; Ferreira Ruiz, 2021; Vecchi and Santos, 2023) and the emergence of a new biological system (for a discussion of this term, see Hao et al., 2021).

It may be of considerable relevance that we and others have previously shown that certain environmental factors, such as hormones, nutrients, drugs, mechanical stress, serum proteins, metabolites, are able to modulate the topography (composition, arrangement) of non-genetic matter, encompassing micelle-like complexes and EVs, as well as the efficiency of their release from donor cells/tissues and their transfer to acceptor cells/tissues (Müller and Müller, 2023c). For example, serum levels of micelle-like GPI-AP complexes have been found to be significantly lower in high-fat fed and genetically predisposed obese rats, as well as in diabetic and obese humans (Müller et al., 2019). This effect was found to be even more pronounced with concomitant hyperinsulinemia and increasing age. The paradoxical decrease in serum levels of the complexes, despite their upregulated release from donor cells/tissues under these specific nutritional/genetic conditions, is due to increased activity of the mammalian serum GPI-PLD (Müller et al., 2020a). The resulting “overcompensation” may prevent harmful consequences of the endocrine transfer of GPI-APs in distant acceptor blood/tissues cells, such as lytic effects of the amphiphilic GPI anchor or activities of GPI-APs at the “wrong” site. Based on the current state of knowledge, the typical characteristic of non-genetic matter encompasses i) transfer from the donor organism, ii) copying in the corresponding organs and tissues of the acceptor organism, the topography of which is susceptible to modulation by environmental factors, and iii) induction of altered phenotypes in the acceptor organism. Since these alterations will reliably recur in the next-generation, processes (i–iii) may be understood as intergenerational inheritance of traits acquired in response to environmental factors.

The detection of DNA as the transforming agent in bacteria about 95 years ago almost immediately led to i) refutation of the old and heavily disputed concept of the inheritance of acquired features, ii) differentiation between inheritance and growth, differentiation as well as development, and iii) exclusion of the existence of any matter of inheritance different from DNA and genes. Now this narrowing should be overcome by inclusion of EVs and micelle-like GPI-AP complexes as non-genetic matter of inheritance. Upon release from donor cells, transfer to and replication by self-organization in acceptor cells, MELs manage to switch their (metabolic) phenotype. Most critical, in rats and humans transfer and topographical alterations of MELs are susceptible to environmental factors. Thus, the mode of action of MELs may be regarded as epigenetic mechanism in the original meaning of the term “epigenetics” which does not rely on DNA or histone modifications, as epigenetics is typically understood at present (for instance, see Feil and Fraga, 2012; Zoghbi and Beaudet, 2016; Tabatabaiefar et al., 2019; Vukic et al., 2019; Bhattarai et al., 2021), contributing to the inheritance of acquired traits. According to the opinion of the authors this option has not been adequately addressed so far, e.g., in studies on the pathogenesis of common complex diseases, in general, and metabolic diseases, in particular.

## Final remarks–novel epigenetics or “entanglements” of heredity

Finally, the view is explained that both intercellular (via both intra- and extracellular paths) and intergenerational transfer of non-genetic matter, encompassing MELs and EVs as well as micelle-like GPI-AP complexes carrying them, complements the repertoire of epigenetic mechanisms (for a review, see Dupont et al., 2012; Feil and Fraga, 2012). The explanation of phenotypic plasticity and the inheritance of acquired traits that is not based on the modifications of DNA or DNA-associated proteins has only recently become accessible to new technologies (e.g., Andrä et al., 2008; Müller et al., 2019). One reason for this is believed to rely on the adherence to the DNA-centric view of heredity that has persisted for almost a century. In fact, that some of the rather unsatisfactory results produced by genome-wide association studies or animal models of disease with over-expressed or inactivated genes (mostly transgenic or “knock-out” mice, respectively) for the identification of (predisposition) genes for diseases, which do not obey Mendelian laws, during the past three decades are presumably due to the restriction to/focus on gene polymorphisms and their complex interplay in connection with the neglect of the possibility of the transfer of topographically (composition, arrangement) altered non-genetic matter (i.e., MELs distorted by environmental factors). At the protein level, too, the few relevant studies, e.g., in the cardiometabolic area, have so far mainly addressed the relationship between hypertension or type II diabetes and structural or functional–possibly epigenetically induced–changes in transmembrane proteins (e.g., Rinaldi and Bohr, 1988; Sanders and Myers, 2004; Desai and Miller, 2018; Saha, 2020), but not in GPI-APs.

It should be instructive to describe the network of human and non-human (f)actors responsible for the exclusion of the transfer of non-genetic matter and acquired traits from the material-discursive practice of studying intergenerational and intercellular inheritance. However, this would require transdisciplinary cooperation between natural sciences and humanities, in general, and genetics, molecular biology, cell biology and “science and technology studies” (STS; for a definition, see above), in particular.

For an approach that aims at radically questioning and shifting those differentiations, i.e., between genetic and non-genetic matter of inheritance, but does not deny them *per se*, it is necessary to soften disciplinary boundaries and allow social and natural science conceptions to meet. “Agential Realism”, a prominent branch within STS, which has been developed for more two decades by the US philosopher, physicist and feminist Karen Barad (2007; for a review, see Hoppe and Lemke, 2018), may be of particular interest in the future investigation of the mutual “entanglements” (Barad, 2010) of the matter of inheritance with many other (human and non-human) (f)actors which together constitute the apparatuses of the production and the observation of heredity phenomenon. For this, texts of philosophy of science and media, sociology and history of science, as well as feminist theory would have to be read through the onto-epistemologic space, built up by genes, DNA, “Gemmules”, “Stirps”, MELs, epigenetics, inheritance of acquired traits, etc., which has been characterized as “diffractive reading” or “jumbling through” by Barad (2010). This necessitates to avoid placing of any of the theories, practices, discourses, and disciplines above the other, quite a formidable challenge.

Characteristic of “Agential Realism” is that differentiations in the production and observation of phenomena (Barad, 2000; Barad, 2003), e.g., between genetic and non-genetic matter, genes and environment, inside and outside of an organism, are not only recognized as being “real by agency” but are even considered to be necessary for coping with contingency and for the creation of classifications and order. However and of uttermost importance, those differentiations must not be regarded as i) being unchangeable, ii) given by “nature” and iii) exclusively socially grounded and constructed, but have to be interpreted as being highly dynamic and flexible, actively produced by a network of (human and non-human) (f)actors. It is this very complex material-discursive practice that must be made comprehensible. In the radical questioning of given, defined, stable, unchangable entities and by analysis of the consequences associated with any introduction of (new) differences, i.e., in the case of inheritance the differentiations between genes, proteins, organelles, membranes, MELs, environmental factors, Karen Barad (2014) shifts the focus to (the emergence and dynamics of) boundaries of phenomena and the entanglements between them.

This questioning of fixed boundaries of phenomena is supported by the concepts of agential cuts, agential apparatuses of the production and observation of phenomena and “intra-actions”. “Intra-action” is a key term of “Agential Realism”. In contrast to the usual interaction, “intra-action” acknowledges that different entities, (f)actors, phenomena, practices, things, matter of inheritance, do not precede the agential cuts and consequently do not “inter-act” as pre-existing units, but rather are produced by them as a consequence, emerging from their “intra-action”.

To make this concept clear, two examples are given: i) Frederick Griffith (1928) produced the famous transforming agent (later identified as DNA by Avery et al., 1944) by first “agentially cutting it apart” from a cultured pathogenic bacterial strain by heating and then “agentially cutting it together” with a non-pathogenic strain. The “intra-actions” prevalent before (in the “smooth” strain) and after the “agential cuts” (in the transformed strain) caused pathogenicity. ii) The distinction between extracellular and intracellular (cytoplasmic) inheritance in the course of the intercellular inheritance of non-genetic matter by SAW biosensing (see Figures 5, 6) requires agential cuts under the involvement by complex networks of human and non-human (f)actors, including researchers and scientific community, the “objects” cultured cells and PMs, and the “subjects” centrifuges, mass spectrometers, culture plates, gel chambers, SAW biosensors, screens, printers, computers. In the course of “agentially cutting” “objects” and “subjects” together, the complex network of these (f)actors may produce reproducible and communicable “traces” or “inscriptions” of the phenomenon which become transformed into data and publications.

Certainly, in most cases it will not be possible to identify all the (f)actors that are involved in the production and observation of a particular phenomenon and could potentially be relevant for the explanation or understanding of its emergence. But this “indeterminacy” should not be misunderstood as an argument for accepting the “given by nature” for the setting of certain agential cuts and the use of certain apparatuses of the production and observation of phenomena, such as in case of Griffith (1928) and genetic (intracellular) inheritance heating for the differentiation between DNA and protein, and in case of Andrä and coworkers (2008), Müller and coworkers (2019 and 2021b) and non-genetic (extracellular) inheritance SAW biosensing for the differentiation between MELs and other cellular constituents. In any case, the usefulness of additional agential cuts, such as cell fractionation and centrifugation, for the identification of non-genetic matter of intracellular non-genetic inheritance has to be considered. In short, the appropriateness of multiple interpretations of quantum physics has long been generally accepted. Why, then, should there be only one interpretation, i.e., only one agential cut, for the phenomenon of heredity?

STS, which have been introduced more than four decades ago by Bruno Latour and Steven Woolgar (Latour and Woolgar, 1979; Latour, 1996), Callon (1986), Donna Haraway (1997) and Joseph Law (2004), should be used to explore the various historical developments, cultural domains and epistemic spaces that contributed to the phenomenon of heredity. François Jacob (2002) has described the transition from procreation to heredity thinking–following Michel Foucault’s approach in Die Ordnung Der Dinge Foucault, (1978)—as a succession of different epistemes separated by sharp epistemological ruptures. The development of theories of inheritance was always dependent on a whole ensemble of cultural contexts, often locally limited, but also extremely divergent. In the long term, they became part of complex configurations of globally distributed technologies and institutions, among them botanical gardens, hospitals, genealogical and statistical archives, plant and animal breeding institutes, (bio)chemical and physiological laboratories, which were by no means in a constant exchange from the beginning, especially not necessarily with regard to the nature of the matter transferred. Moreover, the many conjunctures that had been established along the historical path of the material-discursive practice of inheritance must ultimately be analyzed with regard to the consequences of the foundation of a multitude of nations all over the world, with their centralized bureaucracies, capitalist relations and conditions of industrial production, circulating flows of money, and colonialism.

Importantly, the epistemic space of heredity has not been simply absorbed in theories that served to justify new socio-economic orders. Conversely, there were these new orders themselves in which the epistemic space of heredity emerged again and again. As is only now beginning to be understood, at a time when genetic screening, testing and patents are permeating more and more areas of societal and individual, public and private life, and in which the apparent creative power of genomics, recombinant DNA technologies and synthetic biology is postulated not only to manipulate life but also to create life. Within the past 150 years the epistemic space of heredity has completely reconfigured life itself.

Interestingly, for this seemingly “non-scientific” societal dimension of the concept of heredity, Francis Galton (1876) provided an instructive analogy in his A Theory Of Heredity. His “Stirps” do not represent a static construction or structure, but, as already mentioned, consist of full arrays of dispositions for features that are in a complex flexible mutual interrelationship as well as struggle. In Galton’s opinion, the consequences of this permanent confrontation can best be compared with ‘events of political life’, i.e., those associated with the struggle for space and power, with elections and (political) representation. In this analogy, heredity becomes a distinctly contingent phenomenon. According to Galton, the individual “Gemmules” constituting the “Stirps” reproduce truly themselves. However, which of the many “Gemmules”, he conceived to be contained in the “Stirps”, ‘develop depends on their position in the overall structure’ of the “Stirps”, and whether they are engaged in the resulting interplay of forces against the other “Gemmules” of the “Stirps”. On that basis, Galton explained, for example, the fact that siblings can considerably differ from each other, although one must assume that their respective “Stirps” are quite similar in composition and structure. In analogy to the political and societal spaces, the epistemic microscopic one of the “Stirps” with its elemental dispositions struggling for expression, growth, differentiation and development is juxtaposed to the macroscopic space of the world population, which disintegrates into competing nations, parties, unions, and other groupings. And not surprisingly, there is a relationship between those two spaces in such a way that the dispositions and qualities, that operate in the political space, are predetermined in the “Gemmules” of the “Stirps”. Conversely, shifts in the balance of power in political life will ultimately result in a change in the composition and structure of the “Stirps”. This was precisely the origin and pragmatic “heart piece” of Galton’s program of “positive” eugenics, envisaged to promote the reproduction of the best.

## Data Availability

The datasets generated and analyzed during the current study are available from the corresponding author upon reasonable request and will be provided as the original SAW data files together with the appropriate SAW Inc. software for data visualization and processing (correction and normalization) if required, under consideration of the relevant conditions for licensing of FitMaster, SensMaster, and SequenceMaster.
